# Isolation and molecular characterisation of *Halicephalobus gingivalis* in the brain of a horse in Piedmont, Italy

**DOI:** 10.1186/s13071-017-2070-3

**Published:** 2017-03-07

**Authors:** Maria Domenica Pintore, Francesco Cerutti, Antonio D’Angelo, Cristiano Corona, Paola Gazzuola, Loretta Masoero, Corrado Colombo, Roberto Bona, Carlo Cantile, Simone Peletto, Cristina Casalone, Barbara Iulini

**Affiliations:** 1 Istituto Zooprofilattico Sperimentale of Piemonte Liguria and Valle d’Aosta, Turin, Italy; 2 Department of Veterinary Science, University of Turin, Turin, Italy; 3 Practioner from Turin, Turin, Italy; 4 Department of Veterinary Science, University of Pisa, Pisa, Italy

**Keywords:** Brain, Horse, Meningoencephalitis, *Halicephalobus gingivalis*, Phylogenetics, Zoonosis

## Abstract

**Background:**

A fatal case of meningoencephalitis was reported in a 13-year-old Koninklijk Warmbloed Paard Nederland stallion, suspected of West Nile virus (WNV) infection, in the Piedmont region of Italy. Clinical signs included right head tilt and circling, depression alternated with excitability, fever and lateral strabismus. Combined treatment consisting of dimethylsulfoxide, dexamethasone, sulphonamides and sedative was administered, but because of the poor conditions the horse was euthanatized and submitted for necropsy.

**Results:**

At post-mortem examination no skin lesions were observed, all organs appeared normal on gross evaluation and only head and blood samples were further investigated. Neuropathological findings consisted of granulomatous meningoencephalitis and larvae and adult females of *Halicephalobus gingivalis* were isolated and identified from the digested brain. Frozen brain was submitted to PCR amplification and 220 bp multiple sequence alignment was analysed by Bayesian phylogenetic analysis.

**Conclusions:**

Phylogenetic inference revealed that the isolate belongs to *H. gingivalis* Lineage 3. WN surveillance can help to deepen our knowledge of horse neurological disorders investigating their causes and incidence. Moreover, it can help to understand the geographic distribution of the *H. gingivalis*, to unravel epidemiological information, and to estimate risk for humans.

## Background

*Halicephalobus gingivalis* is a ubiquitous and saprophyte nematode belonging to the order *Rhabditida* and commonly found free-living in association with water, soil, manure and decaying organic matter. Occasionally this parasite affects horses, humans, zebras, cattle and big horn sheep [1–5] generally with a fatal outcome. It is characterized by a rhabditiform esophagus, dorsiflexed ovary and ventroflexed vulva. The pathogenesis is unknown; it is likely the nematode penetrates oral and nasal mucosa or skin lacerations and ulcers, although a possible prenatal, perinatal and transmammary transmission has been suggested in two foals [6]. Moreover, the presence of larvae was demonstrated in urine samples [7], and a fly (*Musca autumnalis*) has been proposed as carrier of larvae [8]. Only eggs, larvae and adult females are found in the tissues involved suggesting a reproduction by parthenogenesis [8].

Infection may be restricted to local lesions or involve multiple organs through the hematogenous and lymphatic spread of the parasite [7, 9]. The most affected organs are brain, kidney, oral and nasal cavities, spinal cord, eye and lymph nodes [1, 7, 9–16]. Occasionally, also legs, adrenal gland, lung, stomach, genitals, mammary gland, pituitary gland and heart [1, 4, 6, 15, 16] can be affected. A definitive diagnosis is possible in vivo by biopsy of the accessible nodular lesions or by post-mortem examination.

## Methods

In August 2012, a 13-year-old Koninklijk Warmbloed Paard Nederland stallion, suspected of West Nile virus (WNV) infection, was presented at clinical examination with a 2-day history of severe and rapidly progressive neurological disorder. The animal was native to The Netherlands but has been residing with other healthy horses in a farm in the Province of Turin, Piedmont region, Italy, since 2004. The horse was vaccinated against Equine herpesvirus 1 (EHV-1).

Clinical signs included fever, depressed mental status alternated with hyperexcitability, right side head tilt, ventro-lateral strabismus and circling. Combined treatment consisting of dimethylsulfoxide, dexamethasone, sulphonamides and sedative was administered, but the horse’s condition continued to deteriorate before it was euthanatized and submitted to necropsy.

At post-mortem, no skin lesions were observed, all organs appeared normal on gross evaluation and only blood and head samples were sent to the Istituto Zooprofilattico Sperimentale of Piemonte, Liguria and Valle d’Aosta in Turin for further investigations. Blood serum was assayed by competitive IgM ELISA kit (Ingenasa Ingezim West Nile) to exclude an early infection with West Nile virus. The brain was sampled and processed as follows: one portion was frozen at -20 °C for biomolecular analyses. The other portion was fixed in 10% neutral formalin buffered, embedded in paraffin, cut at 5 μm and stained by the hematoxylin and eosin (H&E). Portions of frozen brain were digested at 37 °C for 48–72 h with artificial gastric juice composed of 5 g of pepsin and 5 ml of hydrochloric acid diluted in 500 ml of distilled water. A compound microscope and microprojector (Leica-DM LB/DC 300, Wetzlar, Germany) were used to identified and measure the parasites isolated from brain.

DNA was extracted from frozen brain and submitted to PCR amplification of a fragment of the 28S ribosomal rRNA gene using the forward primer #504, 5′-CAA GTA CCG TGA GGG AAA GTT G-3′, and reverse primer #503, 5′-CCT TGG TCC GTG TTT CAA GAC G-3′, according to Nadler et al. [2]. The resulting 262 bp PCR amplicon was then purified and sequenced on an Applied Biosystems 3130 4-capillary DNA sequencer, and manually edited (GenBank acc. no. KY364905). A 220 bp multiple sequence alignment containing 19 *H. gingivalis* sequences and 7 sequences from other genera was built with Clustal Omega v1.2.1 implemented in Seaview 4 software [17]. The most appropriate nucleotide substitution model (HKY + G) was selected by jModelTest2 [18] and used as model input for the Bayesian phylogenetic inference by MrBayes v3.2.6 [19]. The mcmc (Markov chain Monte Carlo) was performed for 1,000,000 generations with two runs of four chains each and the convergence of the runs was assessed with Tracer v1.6. The two mcmc parallel runs converged and reported a posterior probability ESS (effective sampling size) > 200. The phylogenetic tree was visualized with Figtree v1.4.3 and edited for publication with Inkscape v0.91.

## Results

**Fig. 1:**
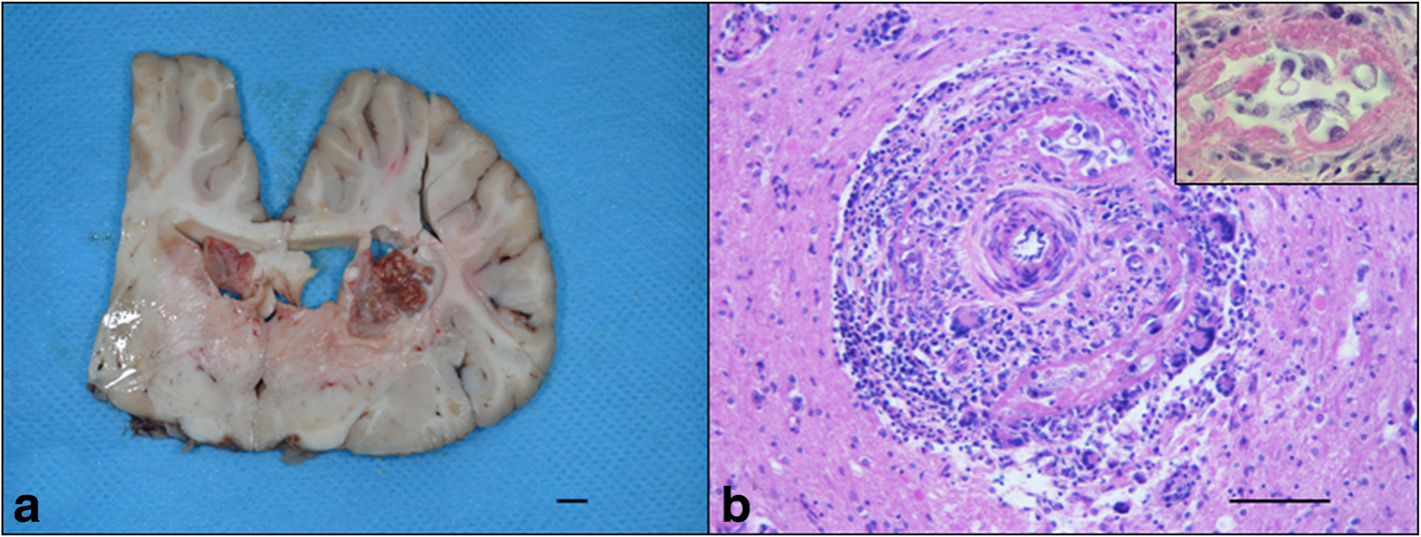
**a** Section of the formalin-fixed brain at the level of the thalamus. Gross appearance of the granulomatous lesions within the lateral ventricles and involving the thalamus on the right side. **b** Brain, thalamus. Angiocentric granulomatous lesion characterized by macrophages, lymphocytes, eosinophils and multinucleated giant cells (hematoxylin-eosin staining). *Scale-bar*: 40 μm. Inset: high magnification of *H. gingivalis* cross sections in the perivascular space (hematoxylin-eosin staining). *Scale-bar*: 10 μm

**Fig. 2:**
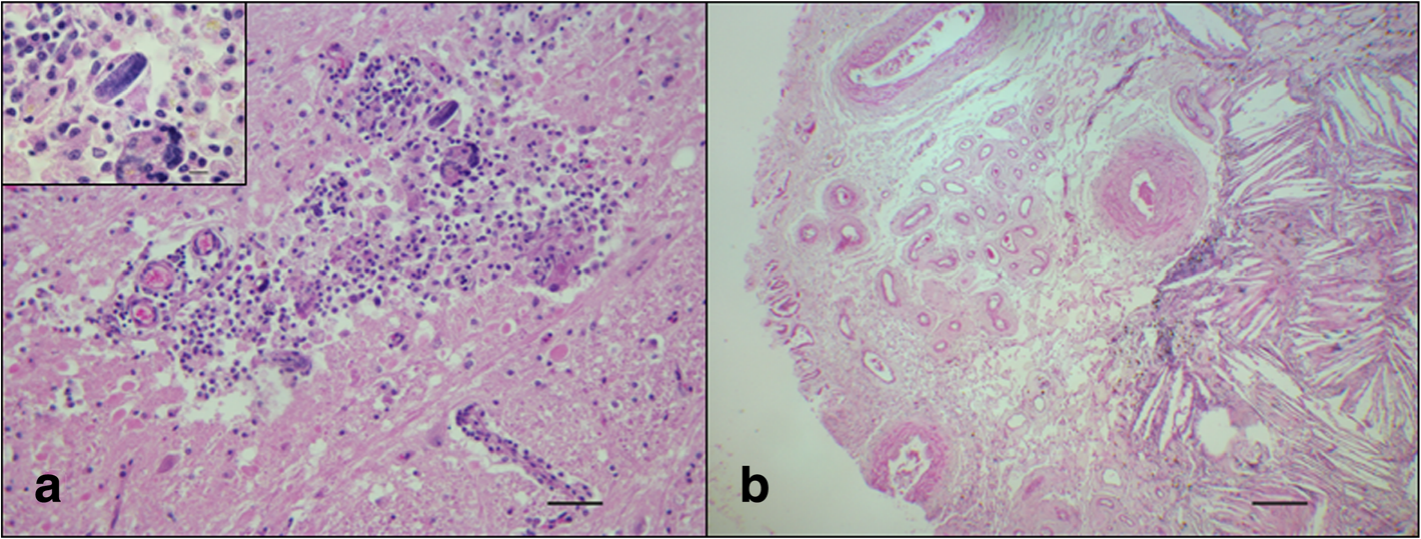
**a** Brain, basal ganglia. Granulomatous inflammation characterized by macrophages, lymphocytes, eosinophils, multinucleated giant cells and many gitter cells. (hematoxylin-eosin staining). *Scale-bar*: 40 μm. Inset: Embryonated egg (hematoxylin-eosin staining). *Scale-bar*: 10 μm. **b** Low magnificaton of cholesterol granuloma. Cholesterol clefts are surrounded by vascular proliferation with hypertrophic walls and scattered foam cells. Fragments of ependymal epithelium are visible (hematoxylin-eosin staining). *Scale-bars*: 300 μm

Macroscopically two space-occupying lesions protruding into the lateral ventricles and adjacent to a malacic area were found at the level of the thalamus (Fig. 1a). The neuropathological findings of the malacic area were consistent with a granulomatous meningoencephalitis predominantly involving the right basal nuclei and thalamus. Many macrophages, lymphocytes, eosinophils and multinucleated giant cells formed perivascular cuffs surrounded by malacic areas infiltrated with many gitter cells. Numerous axonal spheroids were also observed (Fig. 1b). Several nematodes at different stages of development were visible. Larvae and eggs of *H. gingivalis* were dispersed throughout the affected tissue (Fig. 2a). The lesions within the lateral ventricles were well circumscribed and vascularized and were consistent with a cholesterol granuloma (CG). Microscopically, the CG was characterized by a chronic granulomatous reaction to continuous deposition of cholesterol crystals that appeared as empty clefts phagocyted by macrophages (Fig. 2b).

**Fig. 3:**
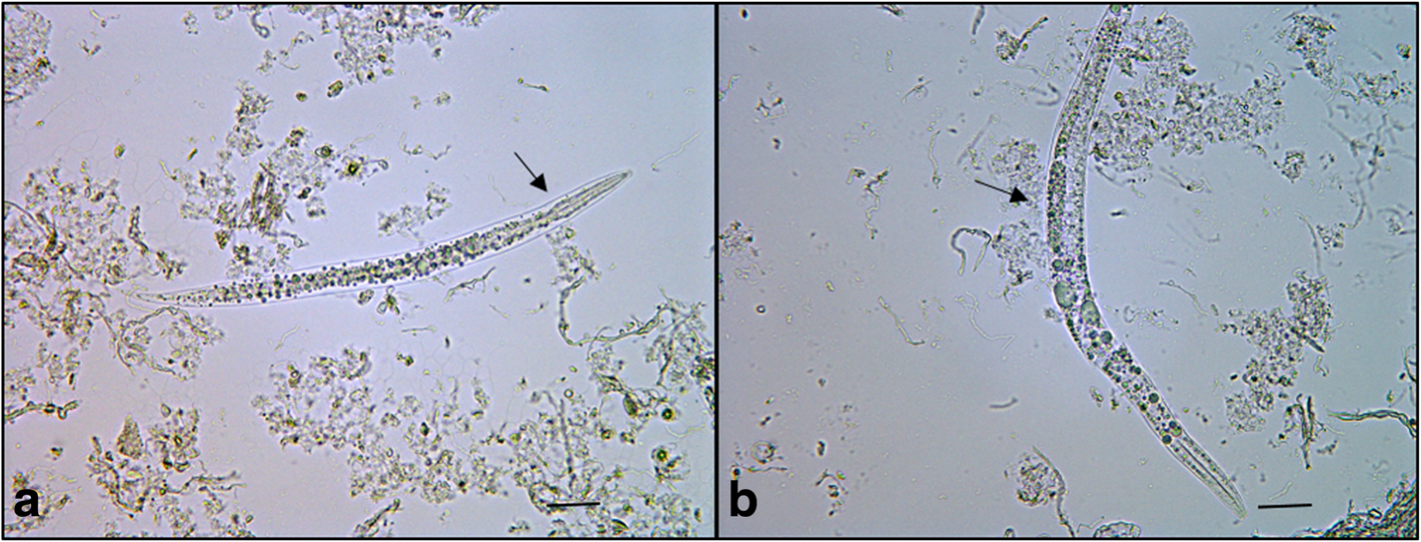
**a** Digested brain. Isolated larva of *H. gingivalis* with rhabdiform esophagus (arrow). **b** Digested brain. Gravid female of *H. gingivalis* with dorsiflexed ovary (arrow). *Scale-bars*: 20 μm

Only larvae and adult females were isolated and identified from the digested brain (Fig. 3a, b) [8]. The parasites were characterized by rhabditiform oesophagus, dorsoflexed ovary and ventroflexed uterus and their average size varied from 250 to 440 μm in length. Serological examination yielded negative results for WNV.

**Fig. 4:**
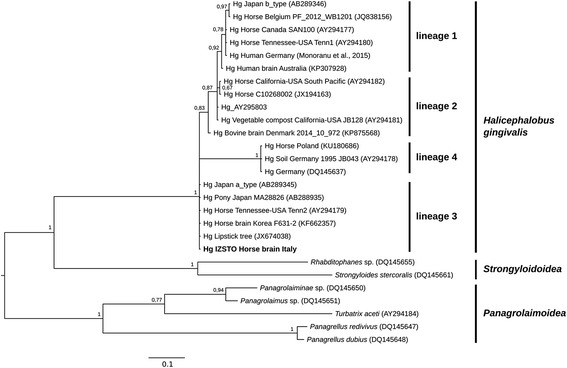
Bayesian inference phylogenetic tree for 19 *H. gingivalis* isolates and 7 outgroup sequences. The node support is expressed as posterior probability, and the scale represents nucleotide substitutions/site. The sample isolated in this work is highlighted in bold (GenBank KY364905)

The *H. gingivalis* 28S rRNA gene was PCR amplified from frozen brain. Analysis of the obtained sequence showed a similarity ranging from 89.5 to 99.7% to 16 homologous sequences representing all the other *H. gingivalis* isolates available in the GenBank database. Posterior probability of the internal nodes well supported all the four lineages previously identified by Nadler et al. [2] and our isolate was included in the Lineage 3 (Fig. 4).

## Discussion

In Italy, there is currently a West Nile monitoring plan in place that requires testing blood and organs of every horse with neurological signs to exclude the virus infection. In this case, WNV was not detected but a diagnosis of granulomatous meningoencephalitis associated with *H. gingivalis* was confirmed by histopathological findings and molecular analysis.

The first case of *H. gingivalis* infection in Italy was reported in 1988 [20] in a horse with renal granulomatous lesion. To date, at least six cases of horse halicephalobiasis have been reported in Italy from different regions [11, 20–23], but no one was characterized by molecular and phylogenetic analysis. To date no human cases have been diagnosed in Italy.

Many cases of horses infected by *H. gingivalis* have been reported in different countries [1, 4, 7–10, 12, 13, 15, 16, 24–33] suggesting a ubiquitous distribution of the nematode. On the other hand, only eight fatal cases of *H. gingivalis* infection have been reported in humans in Canada [34], Australia [35], and Germany [36] and five in the United States [5]. All known human cases presented CNS involvement and neurological symptoms.

Previous studies have demonstrated that geographical distance does not influence the *H. gingivalis* genotype, and specific areas harbour diverse isolates capable of affecting horses [6]. In this case, no apparent skin lesions were visible and the route of infection remains unknown in accordance with other reports [11, 25]. The disease usually shows a rapid progression with granulomatous inflammation and destruction of infected tissues [2]; for this reason, the infection was likely acquired locally. The soil of the farm on which the horse lived was not investigated for the presence of the parasite, and the infection source was not identified. Moreover, both the owner and the referring veterinarian reported that neurological clinical signs have never been observed in this or any other horses on the farm and there was no history of travel outside Piedmont.

*Halicephalobus gingivalis* has a predilection for organs with good blood flow, such as brain and kidney, probably migrating along vascular channels [37]. Clinical signs depend on the location and severity of the lesions, which may lead to the infection easily being confused with other diseases. The most frequent neurological signs are ataxia, depression and abnormal behaviour [20]. Observation of parasite in the lumen and walls of blood vessels suggests that brain involvement probably occurs through hematogenous route [38], but until now it has never been found in blood samples [10]. However, a migration from the eye via optic nerve tract was reported [14].

The brain lesions in our case were like those reported previously [9, 11]. Cholesterol granulomas at the level of the thalamus and lateral ventricles was interpreted as an occasional finding, unrelated with the clinical signs and consistent with the age of the horse. Usually cerebral lesions are diffuse [6] but the most affected areas are cerebellum, thalamus, brainstem and meninges, [1, 6, 9–11, 13–15, 20, 23, 25, 31, 33]. Unfortunately, in this case it was not possible to evaluate the extent of the infection due to the absence of other tissues for examination. Moreover, the cerebral spinal fluid was not examined.

The horse was treated symptomatically but there was no response to therapy. It had not been treated with an anthelmintic drug. To date, therapeutic treatment has been reported to be successful only in a few cases without brain involvement [24, 29, 39], probably because anthelmintic drugs do not cross the blood-brain barrier or due to lacking of sensitivity of *H. gingivalis* to antihelminthic therapies [40]. For this reason, the prognosis is poor and the infection is usually diagnosed during post-mortem examination. To date, there is no *intra vitam* laboratory test, but the examination of urine has been recommended when the infection is suspected [7].

Our study reveals that the *H. gingivalis* isolate belonged to Lineage 3, which includes other isolates from Japan and USA. Unfortunately, only a few sequences are available on public databases, with little information on collection date and location. However, sequence similarity data and phylogenetic analysis confirm that there is no correlation between location and genetics in *H. gingivalis*, based on 28S rRNA gene data. Similarly, there is no apparent correlation between lineages and clinical manifestation of *H. gingivalis*, although the number of available sequences is limited [2]. Notably, the only two *H. gingivalis* sequences available from human cases recently reported in Australia and Germany [35, 36] were both classified by phylogenetic analysis as belonging to Lineage 1. Sequencing of more isolates and the analysis of multiple loci might establish whether a correlation exists between phylogenetic clustering, geographic correlation, clinical signs and the zoonotic potential of *H. gingivalis*.

## Conclusions

Nothing is known about the distribution of *H. gingivalis* in Italy but it could be more widespread than previously thought. In the present report, the meningoencephalitis caused by *H. gingivalis* was diagnosed during WNV surveillance activity as previously reported in Italy in 2012 by Di Francesco and colleagues. This finding emphasizes the role of WNV surveillance in deepening our knowledge of horse neurological disorders investigating their causes and incidence in the country. Moreover, it can help to understand the geographic distribution of the *H. gingivalis*, to unravel epidemiological information, and to estimate risk for humans.

## Abbreviations

AIC: Akaike Information Criterion
BIC: Bayesian information criterion
CG: Cholesterol granuloma
EHV-1: Equine herpesvirus 1
ELISA: Enzyme-linked immunosorbent assay
ESS: Effective sampling size
mcmc: Markov chain Monte Carlo
WNV: West Nile virus
